# Cosmesis after early stage breast cancer treatment with surgery and radiation therapy: experience of patients treated in a Chilean radiotherapy centre

**DOI:** 10.3332/ecancer.2018.819

**Published:** 2018-03-21

**Authors:** Lorena Vargas, Sebastián Solé, Claudio Vicente Solé

**Affiliations:** 1 Radiotherapy Resident, Clínica IRAM, Santiago 7630370, Chile; 2 Faculty of Medicine, Universidad Diego Portales, Santiago 8370068, Chile; 3 Radiation Oncology, Clínica IRAM, Santiago 7630370, Chile; 4 Statistical analysis, Clínica IRAM, Santiago 7630370, Chile

**Keywords:** breast cancer, cosmesis, hypofractionation

## Abstract

**Aim:**

To analyse the overall cosmetic outcome according to patient self-assessment in relation to the fractionation received.

**Materials and methods:**

A questionnaire, drawn up on the basis of subjective rating scales of cosmesis and of acute and late toxicity RTOG/EORTC, EORTC QLQ-C30 (v3) and LENT SOMA, was applied to patients with early-stage breast cancer who received radiotherapy with tangential fields between June 2014 and July 2015. Self-perception of cosmesis, changes in the treated breast, pain and fractionation used (hypofractionation (HF) 42.56 Gy in 16 fractions or conventional fractionation (CF) 50 Gy in 25 fractions) were evaluated.

**Results:**

Three hundred and fifty-two questionnaires were obtained. The median age was 58 years. 45% of patients reported ‘excellent’ cosmesis, 53% ‘good’, and 2% ‘poor’ cosmesis. Cosmesis was considered ‘excellent/good’ by 98% of patients. No statistically significant difference was found in overall cosmesis according to fractionation received (*p* = 0.6).

The most frequent alteration was ‘difference between both breasts’ (77%), and 48% reported change in normal breast colour.

Fifteen percent of patients who are younger than 58 years reported a change of normal breast colour affecting cosmesis compared to 9% of patients older than 58 years (*p* = 0.04).

Complications affecting breast cosmesis were reported by 9% of patients with stages I-II compared with 2% with cancer *in situ* (DCIS) (*p* = 0.04); 14% in stages I-II referred colour change affecting cosmesis compared to 6% of those with DCIS (*p* = 0.03).

Ninety-four percent of patients stated that they would accept treatment again.

**Conclusions:**

No difference in cosmetic results was found between HF and CF in our patients. Great satisfaction regarding cosmetic outcome of cancer treatment was reported, given by 98% of excellent/good cosmesis, and 94% of patients who would receive treatment again.

## Background

Adjuvant radiation therapy after conservative breast surgery represents the standard treatment to improve loco-regional control and survival [1]. Since several studies have demonstrated noninferiority in terms of disease control, survival and toxicity, it is now possible to offer both conventional fractionation radiotherapy (CFRT) and hypofractionated schedules, as they also have the advantage of lower total treatment time [2–5].

The treatment of breast cancer can result in discomfort such as pain and alterations in cosmesis by disturbances in the volume of the breast, scars and skin changes. These changes result from both surgery and adjuvant treatments. Radiation therapy can cause both acute and chronic damages, while the effects of the surgery are usually more immediate [6]. Radiation toxicity can manifest by telangiectasia, oedema and fibrosis of subcutaneous tissue, leading to loss of volume and retraction of the breast. Fibrosis can manifest as induration, to which oedema may also contribute [2].

The study by Whelan *et al* [2] showed no differences in toxicity to skin or subcutaneous tissues at 10 years of follow-up, showing an incidence of late grade 2 toxicity of 5% and 6.4% with CF and hypofractionation (HF), respectively, and 2.7% and 2.5% grade 3, respectively. Also, the START A and B studies showed lower toxicity with hypofractionated schedule, reflected in reduced shrinkage, induration, telangiectasias and breast oedema [4, 5]. Moreover, recent results of a randomized study evaluating acute and 6 months toxicity, and quality of life in patients treated with CFRT compared with HF have been published. The latter group had less fatigue, itching, dermatitis and hyperpigmentation according to the evaluation made by the treating physician [7].

The aim of early-stage breast cancer treatment is not only the oncologic outcome but also the result of cosmesis, which is also a measure of quality of life [8]. Cosmesis and toxicity are usually reported by a physician or a third observer [9, 10]; however, patient self-assessment is of particular interest as well.

The aim of this study is to analyze the overall cosmetic outcome of early stage breast cancer after RT treatment by a patient self-assessment according to the fractionation received.

## Materials and methods

A questionnaire was drawn up on the basis of subjective rating scales of cosmesis [4, 5, 8, 9] also considering the scales of acute and late toxicity RTOG/EORTC, EORTC QLQ-C30 (version 3) and LENT SOMA. From June 2014 to July 2015, this questionnaire was applied to patients diagnosed with early-stage breast cancer treated with conservative surgery, who had received or were to receive adjuvant RT only with tangential fields, with no fields planned to nodal areas, in a Chilean radiotherapy institution. The survey could be applied before the start of RT, at the end of the treatment and/or in follow-up visits by the treating physician or resident. There was no specific interval schedule for follow-up visits.

**Figure 1.**
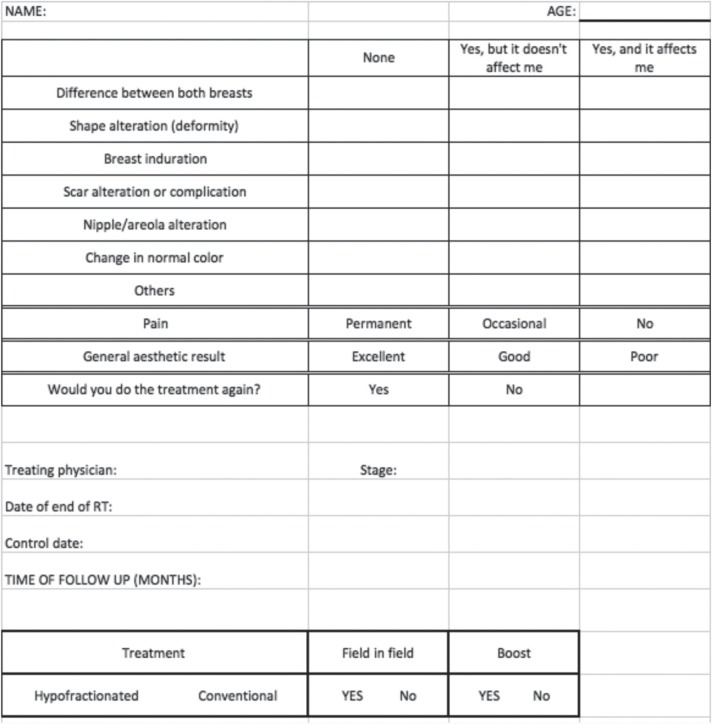
Questionnaire.

Patients were asked to respond according to their own perception if they found differences between the two breasts, if they had any alteration of the form of the treated breast, induration, scar complication, alteration on nipple-areola complex and change in normal colour of the breast. Patients were also asked about the presence of pain, and general aesthetic outcome assessment. Age, stage at diagnosis, fractionation used (HF 42.56 Gy in 16 fractions [2] or CF of 50 Gy in 25 fractions), the use of a surgical bed boost and field in field technique were also recorded. Figure 1 shows the questionnaire used for assessment. All treatment decisions were made in a multidisciplinary tumour board and all patients signed the written informed consent.

A descriptive analysis was performed to calculate proportions, frequencies and medians. Chi square and Kruskal Wallis tests were used for analysis of differences between variables when appropriate. Statistical significance was *p* < 0.05. Analyses were performed in SPSS v21.

## Results

270 patients completed 352 questionnaires between June 2014 and July 2015, patients included were treated from 1999 to June 2015, all patients completed treatment as planned. The median age of patients was 58 years old (31–88) and 24% were younger than 50 years old. Patient, stage at diagnosis and treatment characteristics according to fractionation received are shown in Table 1.

Seventy-one questionnaires were completed at the time of the first visit before radiation treatment, 80 questionnaires at discharge and 201 questionnaires in follow-up visits; 281 questionnaires completed at these two last time points were considered as evaluation of RT treatment effect, 2 were excluded for being incomplete.

Median follow-up was 6 months in both fractionation schedules, and the maximum follow-up was 227 months in a patient treated in 1999. Most questionnaires were completed by patients with less than a year of follow-up (Table 2).

Regarding the overall assessment of cosmesis after RT, 45% (126/279) reported ‘excellent’ cosmesis, 53% (147/279) ‘good’, and 2% (6/279) ‘poor’ cosmesis. Overall cosmesis was considered ‘good/excellent’ by 98% (273/279) of patients.

**Figure 2.**
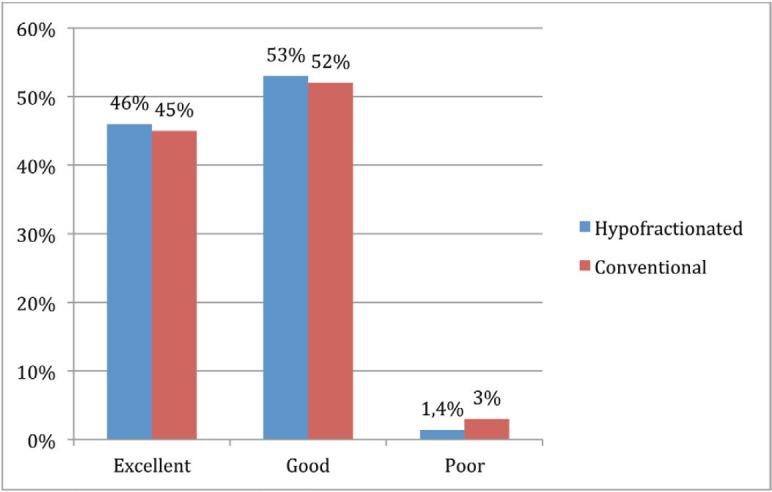
Cosmesis according to fractionation received.

There was no statistical difference in overall cosmesis according to fractionation received (*p* = 0.6) (Figure 2).

No statistically significant difference was found in overall cosmesis regarding the use of boost or field-in-field technique. With regard to the different characteristics interrogated, no statistically significant difference was found in their frequency according to fractionation received.

‘Alteration affecting cosmesis’ and ‘alteration that does not affect cosmesis’ were considered together as the ‘presence of alteration’. In this analysis, ‘difference between both breasts’ was described by 77%, followed by ‘alteration in shape of the breast’ (56%) and then by ‘induration’ (53%). The percentage of patients who reported change in normal breast colour was 48%.

**Figure 3.**
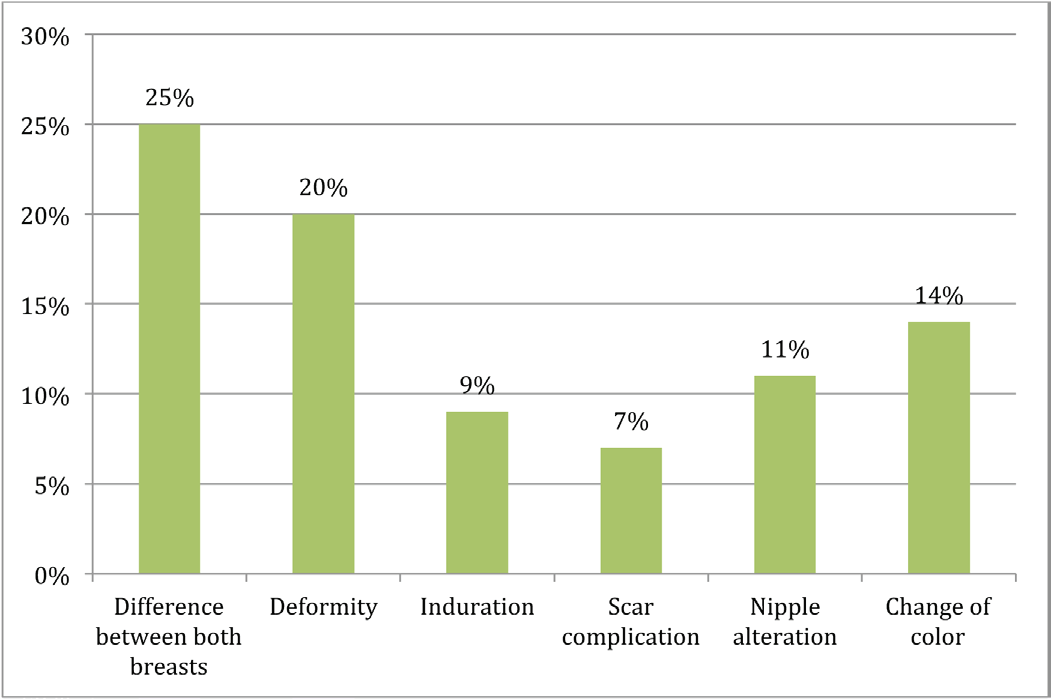
Presence of alteration affecting cosmesis.

**Figure 4.**
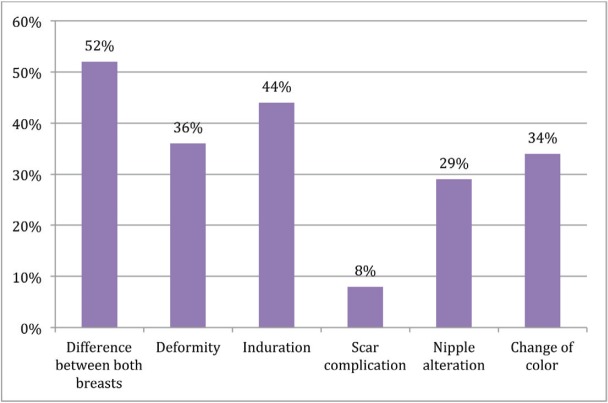
Presence of alteration does not affect cosmesis.

**Figure 5.**
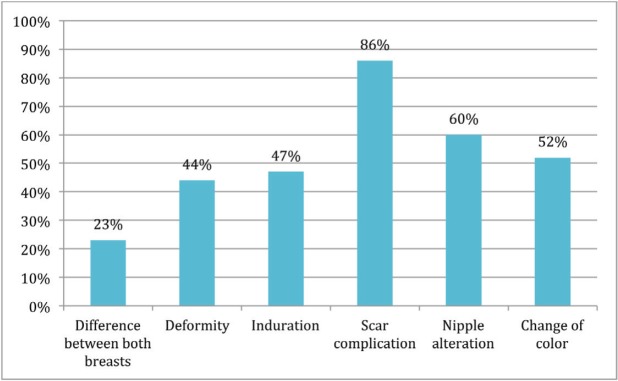
No alteration.

Response distribution for each feature is depicted in Figures 3–5.

In a *post-hoc* analysis, 15% of patients younger than 58 years old reported breast colour change affecting cosmesis compared with 9% of patients older than 58 years old (*p* = 0.04). Patients under 58 years had a greater frequency of breast induration (61% versus 49%, *p* = 0.03).

Nine percent of patients with stages I-II breast cancer reported complications affecting breast cosmesis compared with 2% of patients with ductal carcinoma [ductal carcinoma in situ (DCIS)]. (*p* = 0.04). 14% of patients stage I-II referred colour change affecting cosmesis compared with 6% of those with DCIS (*p* = 0.03).

Pain was reported by 68% (190/279) of the patients. Most of them reported occasional pain (62%, 172/279), whereas only 6% (18/279) reported permanent pain. The rest of the patients reported no presence of pain (32%, 89/279). There were no statistically significant differences in the presence of pain between hypofractionated treatment and conventional treatment (*p* = 0.9).

When considering only the questionnaires before the start of RT and at the end of it, for the variable ‘overall cosmesis’, in both times the most frequent response was good cosmesis (54% at baseline and 64% at discharge), while 3% and 4% reported poor cosmesis at the beginning and at discharge, respectively.

Both before the start of radiation therapy and at the end of it, the frequency described above is maintained, the alteration referred with greater frequency was ‘difference between both breasts’ (66% before and 80% after RT), and in most, without affecting cosmesis. Colour change of the breast upon discharge was reported by 68% (54/80) of the patients and 26% (21/80) claimed that it affected their cosmesis. Thirty-three percent pointed out no change of normal breast colour after RT. Regarding pain, most patients reported occasional pain at the beginning and at the end of RT, no statistically significant difference was found between both time points. Thirty-three patients had questionnaire both before RT and at discharge (Table 3). Finally, 94% of patients stated that they would accept treatment again.

## Discussion

This study demonstrates that aesthetic result after conservative surgery and RT in patients with early-stage breast cancer is generally good as perceived by patients, given by 98% of them who refer excellent/good cosmesis, 46% excellent cosmesis, 52% good and only 2% poor results, which is comparable with data reported in previous literature where excellent or good assessment is described in 66% [10], and in current studies where excellent or good cosmesis is reported in 70% and grade 3 or greater toxicity does not exceed 4% [2]. It has also been reported in other studies with prospective evaluation of cosmesis 93% good–excellent results and only 7% of unsatisfactory aesthetic outcome in patients treated with HF [11].

The alteration most frequently described was difference between both breasts, both globally and before the beginning and at discharge of RT. This is probably the most frequent alteration given the broad meaning that this question may have and it may also be valued differently by each patient. The percentage of patients who reported ‘difference between both breasts’ was 66% before the start of RT, confirming what was mentioned above, sequelae of treatment begin with surgery and then remain or could be increased with adjuvant treatments.

The alteration ‘change in normal breast colour’ affected cosmesis in only a quarter of the patients at discharge, this could be due to management of patient expectations with respect to outcome and treatment toxicity, and that change in colour, either erythema and/or hyperpigmentation, is frequent and usually transient.

There was no statistically significant difference in overall aesthetic result according to the fractionation used, consistent with that reported by other studies [2, 3]. As mentioned previously, studies that evaluated the use of a hypofractionated regimen versus a conventional schedule showed no inferiority in local control but, even more, toxicity was not increased in those patients receiving hypofractionated RT, even when the dose per fraction is greater [2–5]. No difference was found between overall evaluation of cosmesis between the two regimens, with 71% reporting good–excellent cosmesis in the CF group and 70% in HF up to 10 years of follow-up [2]. In other studies of HF, late toxicity was in favour of the hypofractionated regime, considering shrinkage, breast swelling and telangiectasia [3].

The randomized study by Shaitelman *et al* [7] presents data on toxicity between two RT schedules in patients with early-stage breast cancer, treated with conservative surgery and who were to receive RT only to the breast with a tumour bed boost in both groups. Most patients were overweight-obese and over 50% had the maximum dose (Dmax) of 107% or higher relative to the prescribed dose. Acute toxicity was in favour of HF, with lower incidence of grade 2 or greater toxicity (78% in CF versus 47% in HF), less fatigue (13% versus 9%), pruritus (74% versus 50%), breast pain (65% versus 50%), dermatitis (68% versus 36%) and hyperpigmentation (20% versus 9%). At 6 months, there was no difference in toxicity between the two schedules, except in the presence of fatigue, which was higher in the CF group. Although the result of the assessments by the patients themselves is still pending, these data confirm the benefit of HF regarding breast toxicity.

In addition to this, a multicentre, observational study [12], which sought to compare toxicity between HF and CF according to physician and patient assessment, found higher frequency of grade 2 or greater toxicity during treatment as assessed by the treating physician (67% in CF versus 32% in hypofractionated regimen; *p* = 0.003), higher frequency of moderate or severe pain, grade 2 dermatitis, induration, chest wall pain, and moist and dry desquamation. According to the evaluation by the patients, there was greater frequency of pain, moist and dry desquamation, oedema and fatigue with CF [12]. This study represents a more familiar scenario, closer to routine clinical practice setting and closer to the cohort presented in our study, with comparable results to those obtained by us, where hypofractionated RT did not result in worse cosmetic outcome for our patients.

The choice between one fractionation schedule over another in our institution is based, beside disease and patient characteristics, on the capacity of obtaining the best dosimetric parameters possible. The use of modern techniques of 3D conformal RT has led to lower treatment toxicity [13]. In our analysis, most patients were treated with 3D conformal radiation therapy with CT planning, and in half of them field-in-field technique was used to homogenize dose. Boost using was not associated with worse cosmesis, even when higher frequency of fibrosis has been reported in patients receiving tumour bed boost [14]. However in other studies, while it is associated with acute and late skin toxicity, it is not described specifically as a risk factor for worse cosmesis [11]. The use of field-in-field technique was not associated with better cosmesis, probably because patients with worse anatomy required this technique (larger breasts, obese patients, etc.). It could be inferred that field-in-field could counteract the effects of a not ideal anatomy for radiation therapy.

Regarding pain, most patients had occasional pain, both before the start of RT and at the end of it, and the minimum percentage described constant pain. Analgesia requirements were not assessed. Dose inhomogeneity has been described as a predictor of pain after breast RT [15]. The dosimetry data were not collected in the first stage of the study, but it is possible to infer that—based on the good cosmetic results—the small variation in reported pain before and after RT, and the widespread use of field-in-field technique, a homogeneous dosimetry that could explain these results was achieved.

Patient age and tumour size are among the factors affecting cosmetic outcome [11]. In this study, it was found that patients younger than 58 years old reported higher frequency of breast induration, and presented more frequently with changes in the normal colour of the breast that affected cosmesis, while patients with stages I and II breast cancer more commonly referred to any change affecting cosmesis compared with those patients with DCIS. Although tumour size may partially explain these results, we may also consider the effect of surgery on the perception of cosmesis. The effect that axillary surgery had was not evaluated directly, but it may be that those with DCIS who do not have a sentinel node study, and therefore, have less surgical manipulation, have a better evaluation of breast cosmesis and toxicity.

One of the limitations of this study is the subjective nature of the questionnaire that is also not validated. This questionnaire was made based on other scales most widely used in the literature [2–10], although in most the evaluation is done by the physician or by a third-party observer, but not by the patient. In this study, neither a cosmesis analysis by the treating physician or by the third observer, nor objective measurements of aesthetic results such as photographs that allow to quantify changes described by patients were applied. Moreover, it is noteworthy that with photographs alone it is not possible to capture all the changes that can appear in the treated breast, such as induration or pain. Even when there is no objective analysis, it is no less valid to have the perception of our patients relative to the cosmetic outcome of their own treatment, because in addition to oncological outcome the quality of life of our patients cannot be a secondary issue. Finally, it is also possible that there is a selection bias by the treating physician, who could decide whether or not to apply the questionnaire.

## Conclusion

In conclusion, this study found no difference between hypofractionated regimens compared with CF of radiation therapy in our patients in terms of cosmetic results, with no differences in the different variables measured with respect to fractionation received. Great satisfaction regarding the cosmetic outcome of cancer treatment has been reported, given by 98% of excellent/good cosmesis, and 94% of patients who would agree to receive the treatment again.

## Figures and Tables

**Table 1.** Patient and tumour characteristics.

	HF*	CF**
N	127	143
Age (years)		
31–50	6 (5%)	59 (41%)
51–60	44 (35%)	38 (27%)
61–70	44 (35%)	32 (22%)
≥71	33 (26%)	14 (10%)
Tumour Stage***		
0	35 (28%)	27 (19%)
I	67 (53%)	82 (57%)
II	25 (20%)	34 (24%)
Field in field		
Yes	93 (73%)	43 (30%)
No	34 (27%)	100 (70%)
Boost****		
Yes	2 (<2%)	141 (99%)
No	125 (98%)	2 (<2%)

* HF: 42.56 Gy in 16 fractions

** CF: 50 Gy in 25 fractions

*** Tumour stage according TNM AJCC 7^a^ edition

**** Boost: 16 Gy in 8 fractions

Percentages are rounded to whole numbers

**Table 2.** Number of questionnaires at different time points.

Time interval (months)	*N* of questionnaires
Pre—treatment	71
0	80
1–4	55
5–8	46
9–12	10
13–16	13
17–20	17
21–27	14
≥28	46

**Table 3.** Pre-RT and at-discharge responses.

	PRE RADIOTHERAPY			AT DISCHARGE		
	NO	YES, IT DOESN’T AFFECTS	YES, IT AFFECTS	NO	YES, IT DOESN’T AFFECTS	YES, IT AFFECTS
DIFFERENCE BETWEEN BOTH BREASTS	34% 24/71	47% 33/71	20% 14/71	20% 16/80	51% 41/80	29% 23/80
DEFORMITY	58% 41/71	27% 19/71	15% 11/71	40% 32/80	36% 29/80	24% 19/80
INDURATION	37% 26/71	58% 41/71	6% 4/71	35% 28/80	49% 39/80	16% 13/80
SCAR COMPLICATION	86% 60/70	6% 4/70	8% 6/70	81% 65/80	10% 8/80	9% 7/80
NIPPLE ALTERATION	75% 53/71	18% 13/71	7% 5/71	45% 36/80	39% 31/80	16% 13/80
CHANGE OF COLOR	79% 56/71	15% 11/71	6% 4/71	33% 26/80	41% 33/80	26% 21/80
PAIN	31% 22/71	62%* 44/71	7%** 5/71	28% 22/80	63%* 50/80	10%** 8/80

* Occasional pain

** Permanent pain
